# Computed Tomography Appearance of the “Whirlpool Sign” in Ovarian Torsion

**DOI:** 10.5811/cpcem.2021.7.53317

**Published:** 2021-10-05

**Authors:** Joshua K. Livingston, Savannah Gonzales, Mark I. Langdorf

**Affiliations:** * University of California, Irvine, Department of Emergency Medicine, Orange, California; † Cedars-Sinai Medical Center, Department of Obstetrics and Gynecology, Los Angeles, California

**Keywords:** ovarian torsion, computed tomography, ultrasound

## Abstract

**Case Presentation:**

A 28-year-old female presented to the emergency department complaining of right lower abdominal pain. A contrast-enhanced computed tomography (CT) was done, which showed a 15-centimeter right adnexal cyst with adjacent “whirlpool sign” concerning for right ovarian torsion. Transvaginal pelvic ultrasound (US) revealed a hemorrhagic cyst in the right adnexa, with duplex Doppler identifying arterial and venous flow in both ovaries. Laparoscopic surgery confirmed right ovarian torsion with an attached cystic mass, and a right salpingo-oophorectomy was performed given the mass was suspicious for malignancy.

**Discussion:**

Ultrasound is the test of choice for diagnosis of torsion due to its ability to evaluate anatomy and perfusion. When ovarian pathology is on the patient’s right, appendicitis is high in the differential diagnosis, and CT may be obtained first. Here we describe a case where CT first accurately diagnosed ovarian torsion by demonstrating the whirlpool sign, despite an US that showed arterial flow to the ovary. Future studies should determine whether CT alone is sufficient to diagnose or exclude ovarian torsion.

## CASE PRESENTATION

A 28-year-old woman presented to the emergency department with constant right lower quadrant abdominal pain for four days. The patient reported a history of an ovarian cyst about five years earlier, for which she did not seek treatment. Vital signs were normal, and she had right lower quadrant fullness with tenderness. Due to suspicion for appendicitis, a contrast-enhanced computed tomography (CT) was done, which showed a 15-centimeter (cm) right adnexal cyst with adjacent “whirlpool sign” concerning for right ovarian torsion (Image).

Transvaginal pelvic ultrasound (US) then revealed a hemorrhagic cyst in the right adnexa measuring 15 × 9 × 13 cm. Duplex Doppler US of the ovaries identified arterial and venous flow in both ovaries. The presence of a whirlpool sign was not identified on the ultrasound studies. Laparoscopic surgery by obstetrics and gynecology that same night revealed a torsed right ovary with an attached cystic mass and a dusky purple-appearing fallopian tube. Following de-torsion, the fallopian tube was pink and well perfused, and there was no evidence of fallopian or ovarian infarction. However, a right salpingo-oophorectomy was performed because the mass was suspicious for malignancy, with pathology report confirming a mucinous borderline tumor with intraepithelial carcinoma.

## DISCUSSION

Ovarian torsion refers to the complete or partial rotation of the ovary around its ligamentous supports, which can result in partial or complete obstruction of its blood supply.^1^ Ultrasound is the test of choice for diagnosis of torsion due to its ability to evaluate anatomy and perfusion.^2^ When ovarian pathology is on the patient’s right side, appendicitis is high in the differential diagnosis, and CT may be obtained first. In one multicenter, retrospective case-control study of 20 surgically confirmed cases of torsion and 20 controls, there was no significant difference in sensitivity or specificity between ultrasound and CT.^3^ The most common reproducible finding on pelvic US and CT is an adnexal mass, with torsion occurring infrequently in its absence. Other common findings on CT include tube thickening, smooth wall thickening of the twisted ovarian cystic mass, ascites, and uterine deviation to the twisted side.^2^,^4^ Although not often seen, the whirlpool sign of a twisted vascular pedicle in ovarian torsion has been deemed definitive to diagnose torsion on US,^5^ and it is also observed on CT.^4^

Here we describe a case where CT first accurately diagnosed ovarian torsion by demonstrating the whirlpool sign. Despite an US that showed arterial flow to the ovary, suspicion was high based on the CT, and laparoscopy confirmed torsion. This emphasizes that Doppler arterial flow to the ovary does not rule out torsion, especially in the setting of a large adnexal mass as present in this case. Although ovarian torsion remains a clinical diagnosis, future studies should determine whether CT alone is sufficient to diagnose or exclude ovarian torsion.

**CPC-EM Capsule**

What do we already know about this clinical entity?

*Ultrasound is the test of choice for diagnosis of ovarian torsion due to its ability to evaluate anatomy and perfusion.*

What is the major impact of the image(s)?

*This computed tomography (CT) image accurately diagnosed ovarian torsion by demonstrating “whirlpool sign,” despite an ultrasound without evidence of torsion.*

How might this improve emergency medicine practice?

*Computed tomography can be useful in the diagnosis of ovarian torsion. Providers may consider obtaining a CT, particularly when ultrasound is delayed or non-diagnostic.*

## Figures and Tables

**Image.**
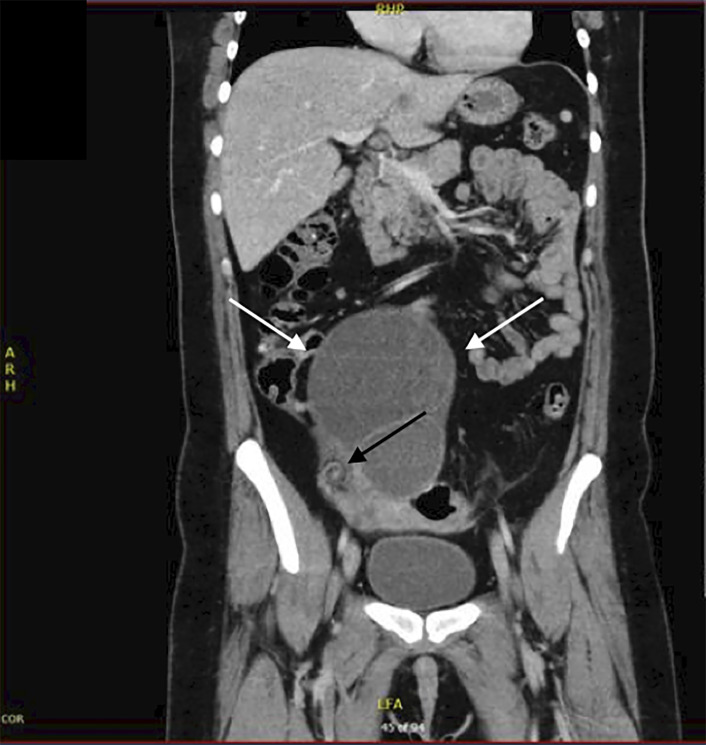
Coronal view of contrast-enhanced computed tomography in a woman with four days of right lower quadrant/pelvic pain, showing the “whirlpool sign” of ovarian torsion (black arrow), confirmed at laparoscopy. Also shown is 10 × 15 centimeter (cm) right ovarian cystic mass (white arrows).
